# Reduced Sodium Current in the Lateral Ventricular Wall Induces Inferolateral J-Waves

**DOI:** 10.3389/fphys.2016.00365

**Published:** 2016-08-26

**Authors:** Veronique M. F. Meijborg, Mark Potse, Chantal E. Conrath, Charly N. W. Belterman, Jacques M. T. De Bakker, Ruben Coronel

**Affiliations:** ^1^Department of Clinical and Experimental Cardiology, Academic Medical CenterAmsterdam, Netherlands; ^2^Interuniversity Cardiology Institute of the NetherlandsUtrecht, Netherlands; ^3^Electrophysiology and Heart Modeling Institute LIRYC, Université de BordeauxBordeaux, France; ^4^Modélisation et calculs pour l'électrophysiologie cardiaque (Carmen) team, Inria Bordeaux Sud-OuestBordeaux, France; ^5^Center for Computational Medicine in Cardiology, Institute of Computational Science, Università della Svizzera italianaLugano, Switzerland; ^6^Department of Medical Physiology, University of UtrechtUtrecht, Netherlands

**Keywords:** J-wave, early repolarization, depolarization, conduction, cellular uncoupling, sodium current

## Abstract

**Background:** J-waves in inferolateral leads are associated with a higher risk for idiopathic ventricular fibrillation. We aimed to test potential mechanisms (depolarization or repolarization dependent) responsible for inferolateral J-waves. We hypothesized that inferolateral J-waves can be caused by regional delayed activation of myocardium that is activated late during normal conditions.

**Methods:** Computer simulations were performed to evaluate how J-point elevation is influenced by reducing sodium current conductivity (G_Na_), increasing transient outward current conductivity (G_to_), or cellular uncoupling in three predefined ventricular regions (lateral, anterior, or septal). Two pig hearts were Langendorff-perfused with selective perfusion with a sodium channel blocker of lateral or anterior/septal regions. Volume-conducted pseudo-electrocardiograms (ECG) were recorded to detect the presence of J-waves. Epicardial unipolar electrograms were simultaneously recorded to obtain activation times (AT).

**Results:** Simulation data showed that conduction slowing, caused by reduced sodium current, in lateral, but not in other regions induced inferolateral J-waves. An increase in transient outward potassium current or cellular uncoupling in the lateral zone elicited slight J-point elevations which did not meet J-wave criteria. Additional conduction slowing in the entire heart attenuated J-waves and J-point elevations on the ECG, because of masking by the QRS. Experimental data confirmed that conduction slowing attributed to sodium channel blockade in the left lateral but not in the anterior/septal ventricular region induced inferolateral J-waves. J-waves coincided with the delayed activation.

**Conclusion:** Reduced sodium current in the left lateral ventricular myocardium can cause inferolateral J-waves on the ECG.

## Introduction

J-waves in inferolateral leads of the surface electrocardiogram (ECG)—or early repolarization (ER) pattern—are characterized as an elevation of the QRS-ST junction manifested as a notch or slur (Sacher et al., 2013; Mizusawa and Bezzina, 2014; Macfarlane et al., 2015). The J-wave was considered a benign phenomenon (Shipley and Hallaran, 1935) until Haïssaguerre et al. demonstrated an increased prevalence of J-waves in patients with idiopathic ventricular fibrillation (Haïssaguerre et al., 2008). This association was confirmed by a meta-analysis of nine studies (Wu et al., 2013).

The mechanism underlying the inferolateral J-waves—or ER pattern—is subject of an ongoing debate (Wellens, 2008; Hoogendijk et al., 2013). Yan and colleagues proposed a cellular mechanism for J-waves based on experiments performed in canine arterially perfused ventricular wedge preparations (Yan and Antzelevitch, 1996). They postulated that J-waves are generated by a transmural voltage gradient resulting from a more prominent transient outward potassium current (I_to_) in the sub-epicardium, leading to a more prominent action potential (AP) notch than in the sub-endocardium. An alternative mechanism is based on regional conduction slowing. Late potentials at the terminal QRS complex in the ECG have been related to delayed activation (Simson et al., 1983). Because of the analogy between inferolateral J-waves and the ST segment elevations in the Brugada Syndrome some investigators posit a common mechanism for the two syndromes (Antzelevitch and Yan, 2010). However, sodium channel blockers cause a differential effect, as these are used to provoke ST segment elevations in the right precordial leads in Brugada Syndrome, but attenuate inferolateral J-waves (Roten et al., 2012). Also, the different location of the J-wave or ST segment elevations—right precordial (Antzelevitch et al., 2005) or inferolateral leads (Haïssaguerre et al., 2008)—indicates involvement of different regions. The inferolateral location of J-waves suggests a substrate in the inferolateral area of the heart, which is normally a late activated region (Durrer et al., 1970). We surmise that when this area undergoes additional conduction slowing the delayed AP will generate a voltage gradient just strong enough to cause a J-wave in the inferolateral leads.

The aim of this study was (1) to test whether delayed depolarization and/or early repolarization can cause J-waves, (2) to test whether left lateral involvement is essential for J-wave appearance in inferolateral leads, and (3) to evaluate a mechanism by which sodium channel blockers can reduce J-waves. For these purposes we used a computational approach. We also employed a pig model to replicate the computational findings on regional sodium channel blockade. We selected a pig model to test the conduction hypothesis because pig hearts lack I_to_ (Li et al., 2003), which could therefore not have interfered with the changes in activation.

## Materials and methods

### Computer simulations

**Figure 1:**
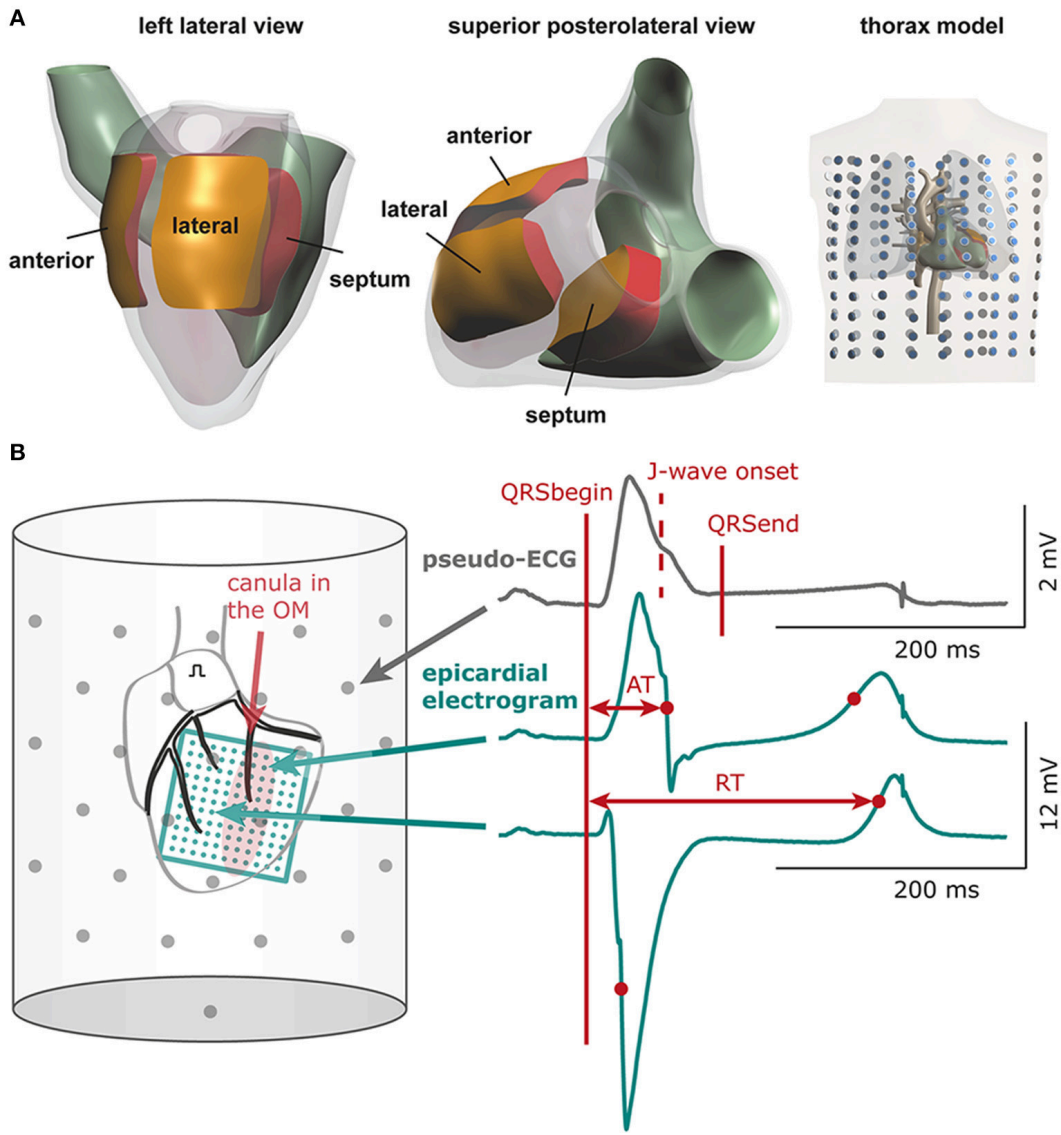
**Computational and experimental setup**. **(A)** Heart and thorax model with indication of the three areas in which electrical properties were varied: lateral, anterior, and septal zone. Dots in thorax model indicate electrode positions for which surface ECGs were calculated **(B)** Schematic of experimental setup. The bucket wall contained 61 electrodes (*gray dots*). QRSbegin and QRSend mark the maximum QRS duration determined using all leads and may deviate from the QRS duration in a single lead. The 11 × 11 electrode grid (*green*) overlies the cannulated left obtuse marginal coronary artery (OM, red shaded area: selectively perfused tissue). See Supplementary Figure S1 for electrode configuration of the 9 × 12 grid. AT, activation time; RT, repolarization time.

A detailed description of the computer model is provided in the Data Supplement. Computer simulations were performed to test the possible contribution of three different electrical properties in the genesis of inferolateral J-waves (or ER-pattern). Within the modeled heart three areas were defined: lateral zone, anterior zone, and septal zone (Figure 1A). Within each area we simulated the following interventions and evaluated their effects on the ECG. By reducing the sodium current conductivity (G_Na_) to 12.5% of baseline condition we tested the depolarization hypothesis, whereas by increasing the transient outward potassium current conductivity (G_to_) 10-fold we tested the repolarization hypothesis. As an alternative test for the depolarization hypothesis we simulated diffuse fibrosis with consequent conduction slowing by reducing the intracellular and extracellular conductivity to 12.5% of baseline condition (cellular uncoupling). The factor of reducing the G_Na_ to 12.5% was chosen by a stepwise reduction of G_Na_ to ½, ¼, ⅛, and 1/16 and selecting the largest value at which the effects of J-point elevations were present. Similarly, the increase of G_to_ was selected by a stepwise increase of G_to_ to 5, 10, and 15 ×, whereby the smallest increase to produce J-wave elevation was chosen. We also exaggerated the simulation to a 20-fold reduction of G_Na_ (= 5% of baseline) and a 20-fold increase of G_to_ in order to evaluate the increment of pathophysiology, which may occur preceding an arrhythmic event (Haïssaguerre et al., 2008). On top of the simulations that induced the most and largest J-waves or J-point elevations, we tested the effects of “systemic” sodium channels blockers by reducing G_Na_ outside the affected zone to 40% of baseline. The 40% was chosen as the value in which J-waves or J-points disappeared.

In the calculated 12-lead ECGs we determined the *total QRS duration* (first QRS onset in any lead to last QRS end in any lead), J-point amplitude, and presence of J-point elevations and J-waves. The *J-point (Jp)* was defined as the top of the end QRS notch or as the point where end QRS slurring started according to the consensus report (Macfarlane et al., 2015). A *J-point elevation* was defined as a Jp amplitude of 0.05 mV or more in an inferolateral lead (I, II, III, aVF, aVL, and V4-V6). A *J-wave* was a J-point elevation (notch or slur) of 0.1 mV or more. *Difference ECGs* were obtained by subtracting baseline ECGs from intervention ECGs.

### Experimental setup

The experimental protocol was approved by the local Animal Experiments Committee (Academic Medical Center, University of Amsterdam) and carried out in accordance with national and institutional guidelines.

Pigs (*n* = 2, male, 50–60 kg) were premedicated, intubated, and ventilated. The heart was excised and perfused according to Langendorff with a (circa 1:1) blood-Tyrode's mixture (pH = 7.35–7.45). The left obtuse marginal coronary artery (OM) or left anterior descending coronary artery (LAD) was separately cannulated. See Data Supplement for more details. The cannula was connected to a separate temperature-controlled perfusion system with the same blood-Tyrode's mixture and with a side branch for the infusion of flecainide (Tambocor, 3M Nederland, Zoeterwoude, The Netherlands, 6 or 60 μM). After baseline electrophysiological recordings, we administered flecainide to the OM/LAD cannula. For the OM perfusion the concentration was 60 μM and for LAD perfusion this was 6 μM. These are calculated concentrations based on the circulating volumes in the two perfusion systems. Initially we used a high concentration. This was chosen because the recirculating system limits the time during which a drug can be infused regionally without entering the main circulating system and causing conduction slowing in the entire heart (this is a characteristic of the cardiac circulation). During the wash-in of the drug temporary intermediate concentrations are present. During this wash-in phase the observations were made at a similar degree of conduction slowing. With the infusion of a high flecainide concentration spontaneous arrhythmias occurred after a couple of minutes following the measurements. Therefore, we used a lower concentration in the following experiment and we waited until a similar activation delay occurred in the myocardial regions. Data analysis was restricted to the conditions in which a similar degree of conduction slowing was present, and therefore a similar degree of sodium blockade (independent of the final concentration of flecainide).

### Electrophysiological recordings

The left atrium was paced at a cycle length of 450 ms. An 11 × 11 electrode grid (OM perfusion) or a 9 × 12 electrode grid (LAD perfusion) was fixed to the epicardial surface overlapping the entire LV (and with the 9 × 12 grid also the anterior RV) to obtain local unipolar epicardial electrograms. Supplementary Methods and Supplementary Figure S1 provide details on the electrode configurations. The Langendorff-perfused heart was submerged in a bucket filled with perfusion fluid, containing 61 electrodes to obtain pseudo-electrocardiograms (pseudo-ECGs, Figure 1B). The reference signal was the average of all electrodes.

In the pseudo-ECGs we determined the *maximum QT interval* and *maximum QRS duration* including the J-wave. Definition for Jp was similar as described above. Because the pseudo-ECG amplitudes were about twice those of real ECGs we adjusted the J-wave criteria accordingly. J-point elevations ≥0.2 mV were denoted as *J-waves*. *J-wave onset (Jo)* was defined as time of first deviation at the end of QRS complex initiating a J-wave—notch or slur—relative to QRS onset (Macfarlane et al., 2015). The inferolateral leads constituted the six columns of electrodes opposite the LV area (*n* = 18) and one bottom electrode (gray boxes in **Figure 5**). *J-wave interval* was defined as Jo to end of QRS. In the *difference ECG*—flecainide ECG minus baseline ECG—we determined the *moment* and *amplitude* of maximum peak difference (positive/negative). In each local unipolar electrogram we determined activation times (ATs) and repolarization times as before (Figure 1B; Coronel et al., 2006). *Difference AT maps* were calculated as flecainide AT map minus baselines AT map. Recordings with ST segment elevation or a flat T-wave were excluded from analysis of repolarization times. Signal analysis was performed offline using software (Potse et al., 2002) based on Matlab (The MathWorks, Inc., Natick, MA, USA).

### Statistics

Continuous variables were presented as mean ± SD if normally distributed and as median (25th–75th percentile) if not normally distributed.

## Results

### Simulations

Table 1 summarizes the simulation data on QRS durations, J-wave occurrence, maximum activation time, and activation delay for each zone of the various simulations. The ultimate AT in the anterior and lateral zones were later than in the septal zone.

**Table 1:** **Simulation data**.

	**QRS (ms)**	**AT max (ms)**	**AT delay (ms)**	**J-wave**		**Difference ECG**	
				**Amax (mV)**	**#leads (Jp ≥ 0.1 mV (Jp ≥ 0.05 mV))**	**Apeak (mV)**	**Tpeak (ms)**
**Baseline**	81	–	–	0	0 (0)	–	–
Lateral		61					
Anterior		64					
Septal		49					
**G_Na_ Reduction**							
Lateral	94	93	14	0.14	4 (5)	0.40	54
Anterior	88	88	13	0.10	1 (1)	0.35	58
Septal	81	65	11	0	0 (0)	0.33	38
**G_to_ Increase**							
Lateral	99	61	0	0.07	0 (4)	0.13	57
Anterior	113	64	0	0.09	0 (3)	−0.15	61
Septal	99	51	1	0	0 (0)	0.21	47
**Uncoupling**							
Lateral	96	103	15	0.06	0 (2)	−0.27	54
Anterior	94	98	14	0.09	0 (2)	0.20	51
Septal	82	83	0	0	0 (0)	0.23	45

*QRS, QRS duration; AT max, maximum activation time in affected zone; AT delay, activation time delay in affected zone; A max, maximum J-point amplitude; # leads, number of leads with J-waves; Apeak, amplitude of maximum peak in difference ECG; Tpeak, timing of maximum peak in difference ECG*.

### Simulations of regional G_Na_ reduction

**Figure 2:**
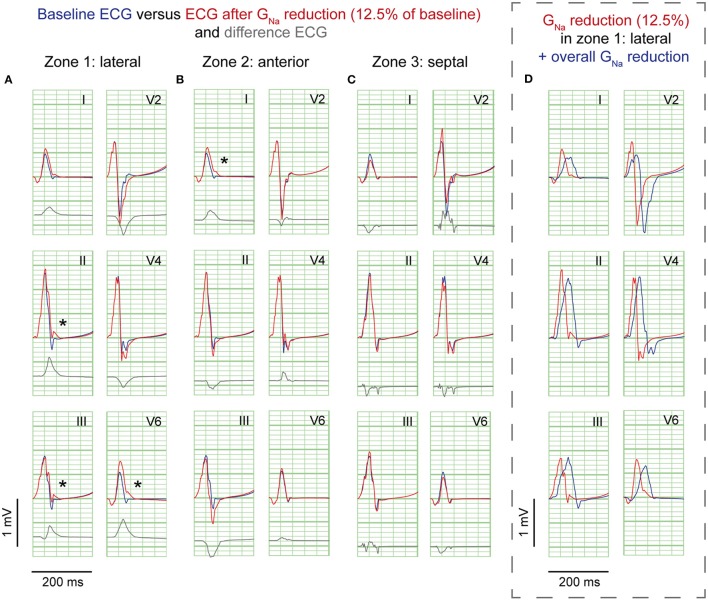
**G_**Na**_ reduction simulations**. ECG at baseline (*blue*) and resulting from G_Na_ reduction to 12.5% of baseline (*red*) in 3 zones; **(A)**, lateral; **(B)**, anterior; **(C)**, septum. *Gray*, Difference ECG (G_Na_ reduction minus baseline). Asterisks, J-waves. *Boxed panel* **(D)**, additional G_Na_ reduction in the rest of the heart (*blue*) causes disappearance of J-waves.

G_Na_ reduction to 12.5% in the affected region caused a delay of the AP without affecting the AP morphology (Supplementary Figure S2A). Figure 2 shows 6 ECG leads at baseline and after G_Na_ reduction in each zone.

G_Na_ reduction in the lateral zone led to J-waves in the inferolateral leads (II, III, aVF, and V6) with a maximum amplitude of 0.14 mV in lead II (Table 1). The extremity leads showed J-wave notches and lead V6 a J-wave slur. G_Na_ reduction in the anterior zone led to a notching J-wave in lead I only, and to J-point depressions in the inferior leads (II, III, aVF). G_Na_ reduction in the septal zone did not induce J-waves.

To exclude a secondary role of I_to_ for J-wave induction in these simulations, we performed a G_Na_ intervention in the lateral zone in a model lacking I_to_ (G_to_ = 0 and G_Na_ = 12.5% of baseline). In this model, the reduction of G_Na_ in the lateral zone caused similar results in J-point elevations albeit with circa 0.02 mV lower amplitudes, which can be explained by the small differences in QRS morphology between the reference ECGs of the models with and without I_to_ (Supplementary Figure S3). When G_Na_ was amply reduced to 5% of baseline in this I_to_ lacking model, J-wave amplitudes were increased about 2-fold compared to the model with G_Na_ reduction to 12.5% (Supplementary Figure S4A).

### Simulations of regional G_to_ increase

**Figure 3:**
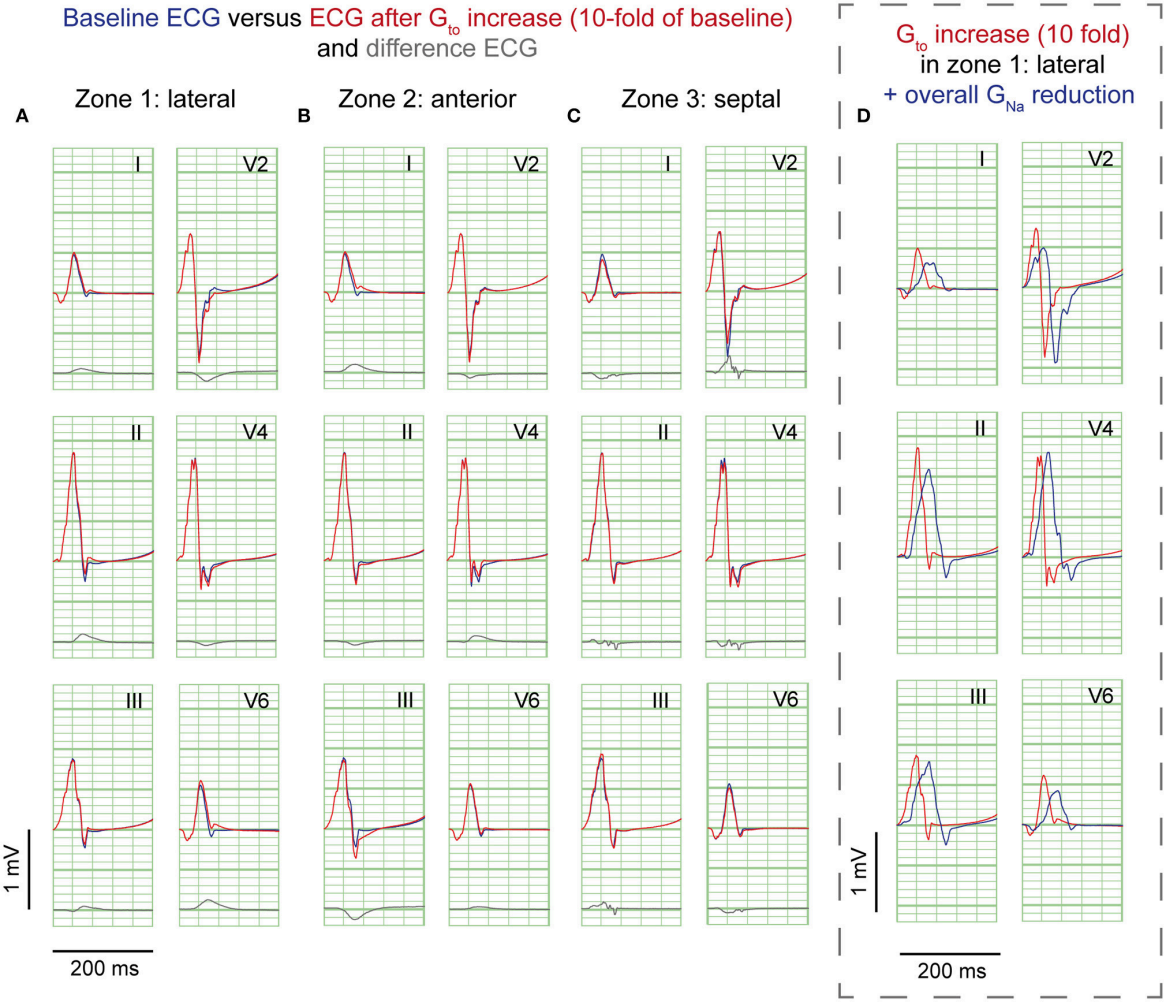
**G_**to**_ reduction simulations**. ECG at baseline (*blue*) and resulting from a 10-fold G_to_ increase (*red*). No J-waves emerge after G_to_ increase. Organization as in Figure 2.

A 10-fold G_to_ increase caused a deeper AP notch at the epicardium of the affected region, without influencing the endocardial AP notch (Supplementary Figure S2B). Figure 3 shows the ECG results of a 10-fold G_to_ increase in each zone. In all simulations of G_to_ increase J-waves were absent. There were J-point elevations, although in fewer leads, and with lower amplitudes compared to G_Na_ reduction (Table 1). Overall, G_to_ increase did prolong the QRS duration, but did not delay activation anywhere. When G_to_ was amply increased to 20-fold of baseline (until loss of AP dome occurred), J-point amplitudes increased about four times followed by ST-segment elevation in the inferolateral leads (Supplementary Figure S4B) compared to a 10-fold G_to_ increase. The ECG changes during a G_to_ increase did not resemble the typical inferolateral J-wave pattern that has been associated with idiopathic ventricular fibrillation (notch or slur in leads I, II, III, aVF, aVL, and V4-V6, see methods).

### Simulations of regional cellular uncoupling

**Figure 4:**
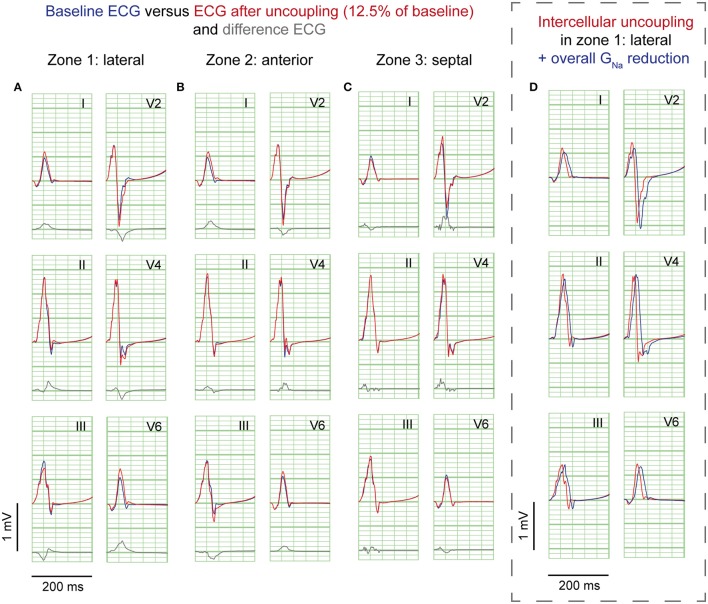
**Cellular uncoupling simulations**. ECG at baseline (*blue*) and resulting from cellular uncoupling to 12.5% of baseline (*red*). No J-waves emerge after uncoupling. Organization as in Figure 2.

Cellular uncoupling (i.e., reduction of intracellular and extracellular conductivity to 12.5% of baseline) in the affected region caused a delay of the AP without affecting the AP morphology (Supplementary Figure S2C). Figure 4 shows the ECG results of cellular uncoupling in each zone. By reducing intercellular coupling in the lateral or anterior region, small J-point notching was induced but no J-waves appeared. The difference ECGs were of intermediate amplitude compared to G_Na_ reduction and G_to_ increase. Timing of the difference ECG peak was similar as with G_Na_ reduction and G_to_ increase. Cellular uncoupling in the lateral and anterior region induced activation delays of about 15 ms and caused maximum activation in the affected zone that determined the end of QRS (Table 1).

### G_Na_ reduction in the rest of the heart on top of J-waves and J-point elevations

Sodium channel blockers can attenuate inferolateral J-waves (Roten et al., 2012). Therefore, we simulated a G_Na_ reduction in the rest of the heart on top of each intervention in the lateral zone (last column in Figures 2–4). In the 3 simulations with G_Na_ reduction in the rest of the heart, pre-existing J-waves or J-point elevations shrunk or disappeared, masked by the QRS complex that widened with 27, 22, and 17 ms (G_Na_ reduction, G_to_ increase, and cellular uncoupling, respectively). G_Na_ reduction in the rest of the heart delayed activation in all zones with latest activation occurring outside the 3 zones (i.e., 116 ms in the G_Na_ reduction and G_to_ increase interventions) or in the lateral zone (i.e., 123 ms in the cellular uncoupling intervention).

### Experiments

**Figure 5:**
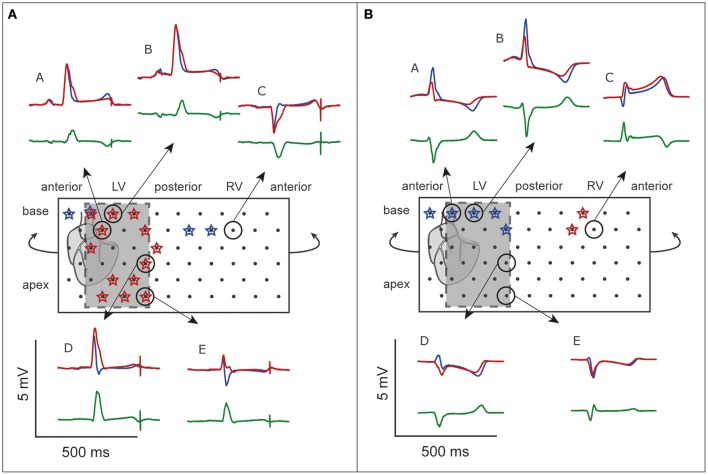
**ECG before and after flecainide**. Unfolded bucket (middle boxes) with electrodes (*black dots*) and heart position. *Shaded boxes* indicate the inferolateral area. **(A)**, OM perfusion. **(B)**, LAD perfusion. *Stars* indicate leads showing J-waves. Surrounding are examples of pseudo-ECGs (*blue*, baseline; *red*, during flecainide). *Green*, difference ECG. A and E correspond with the pseudo-ECGs shown in Figures 6, 7. Flecainide infusion induced or exacerbated inferolateral J-waves in the OM perfusion but not in the LAD perfusion.

Figure 5 shows the unfolded bucket with electrodes and some examples of pseudo-ECGs at baseline and during OM flecainide infusion (60 μM). In this heart, J-waves appeared on the inferolateral pseudo-ECG leads. One J-wave was observed just outside this area, with reciprocal J-point depressions in the other leads (Figure 5: pseudo-ECG at C). At baseline J-waves were present in 4 leads. After flecainide infusion the number of J-waves in inferolateral leads increased, while in the other leads 2 of 3 J-waves disappeared (Table 2: OM perfusion). The amplitude of the difference ECG was larger and positive in inferolateral leads compared to the other leads. After flecainide infusion the QRS duration was increased by 26 ms due to arising J-waves. In the heart with LAD perfusion (Figure 5, Table 2), some J-waves were present at baseline, albeit mainly in the most superior leads. After flecainide infusion (6 μM) all J-waves disappeared in inferolateral leads and arose in 2 leads near the RV posterior wall. The amplitude of the difference ECG was negative and larger in inferolateral leads than in the other leads (Table 2: LAD perfusion). The peak of the difference ECG also occurred earlier during LAD perfusion [15 (12–27) ms] compared to OM perfusion [35 (28–41) ms].

**Table 2:** **ECG characteristics at baseline and during flecainide**.

	**OM perfusion**		**LAD perfusion**	
	**Baseline**	**Flecainide (60 μM)**	**Baseline**	**Flecainide (6 μM)**
Total QRS, ms	87	113	77	76
**INFEROLATERAL LEADS, *n* = 19**				
J-wave # leads, N/n	1/19	10/19	4/19	0/19
Jo, ms	62	42	45	-
A_dECG, mV	0.22 (0.14–0.36)		−0.25 (−0.35—0.20)	
**OTHER LEADS, *n* = 42**				
J-wave # leads, N/n	3/42	1/42	1/42	2/42
Jo, ms	55	55	48	34
A_dECG, mV	−0.11 (−0.17—0.05)		0.17 (−0.16—0.23)	
QT interval, ms	294	300	273	269

*QRS, QRS duration; J-wave # leads, number of leads with J-wave (N) out of number of selected leads (n); Jo, first onset of J-wave; A_dECG, maximum or minimum amplitude of difference ECG [median (25th–75th percentile)]*.

### Activation maps

**Figure 6:**
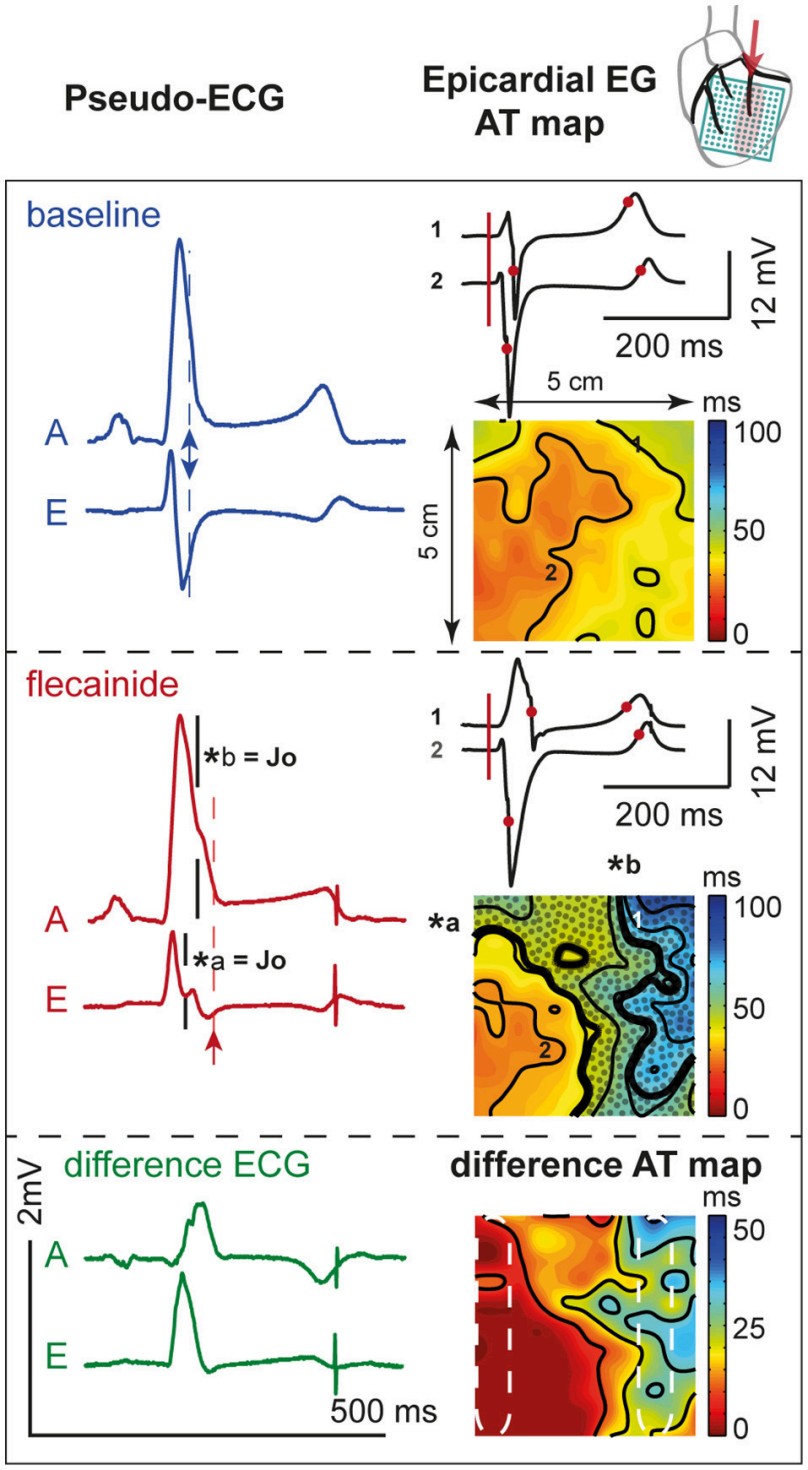
**Activation during**. OM perfusion. Two pseudo-ECGs **(left column)**; activation (AT) maps (isochrone lines at 10 ms intervals) with two epicardial electrograms (EG, **right column**) at baseline **(top panel)** and after 60 μM flecainide infusion **(middle panel)**. AT map: *thick black lines* indicate isochrones of J-waves (^*^a and ^*^b). *Dotted area* represents overlap with J-wave interval, which outreaches the AT map. Numbers *1* (“perfused” region) and *2* (“not perfused” region) recording sites of the corresponding EGs. Pseudo-ECG: arrows indicate the moment of last activation in the AT map. *A* and *E* correspond with pseudo-ECGs in Figure 5. **Bottom panel:** shows the difference pseudo-ECGs and difference AT map. Dashed white ellipses indicate a region within and outside the area perfused with flecainide used to determine mean ATs. Note that the effect of flecainide extended beyond the QRS duration before application and that the region subjected to conduction slowing (right side of AT map) was late activated before application of flecainide.

Figure 6 demonstrates the activation maps at baseline and during flecainide infusion (60 μM) in the OM. At baseline, the OM region was activated latest (right side of the AT map), and showed the largest conduction delay after flecainide infusion. The J-wave interval overlaps this region of delayed activation (dotted surface in flecainide AT map) and even outreaches the AT map. We quantified the activation delay by selecting a column of electrodes inside and outside the OM region (white striped boxes) and summarized the data (Table 3). After flecainide infusion the delay in AT was larger in the OM region than in the rest of the LV. In the non-perfused regions no relevant changes occurred, as expected.

**Table 3:** **Data of local electrograms**.

	**Perfused**		**Not perfused**	
	**Baseline mean ± SD**	**Flecainide mean ± SD**	**Baseline mean ± SD**	**Flecainide mean ± SD**
**OM PERFUSION**				
No. electrodes	10	10	10	10
AT, ms	39 ± 3	67 ± 9	31 ± 10	34 ± 11
AT delay, ms	–	28 ± 7	–	3 ± 3
RT, ms	236 ± 7	239 ± 5	248 ± 5	247 ± 4
**LAD PERFUSION**				
No. electrodes	8	7	8	8
AT, ms	26 ± 10	42 ± 16	31 ± 5	28 ± 5
AT delay, ms	–	16 ± 6	–	−3 ± 1
RT, ms	214 ± 4	234 ± 15	220 ± 7	209 ± 9

*“Perfused” and “not perfused” refers to region that is or is not selectively perfused. AT, activation time; RT, repolarization time*.

**Figure 7:**
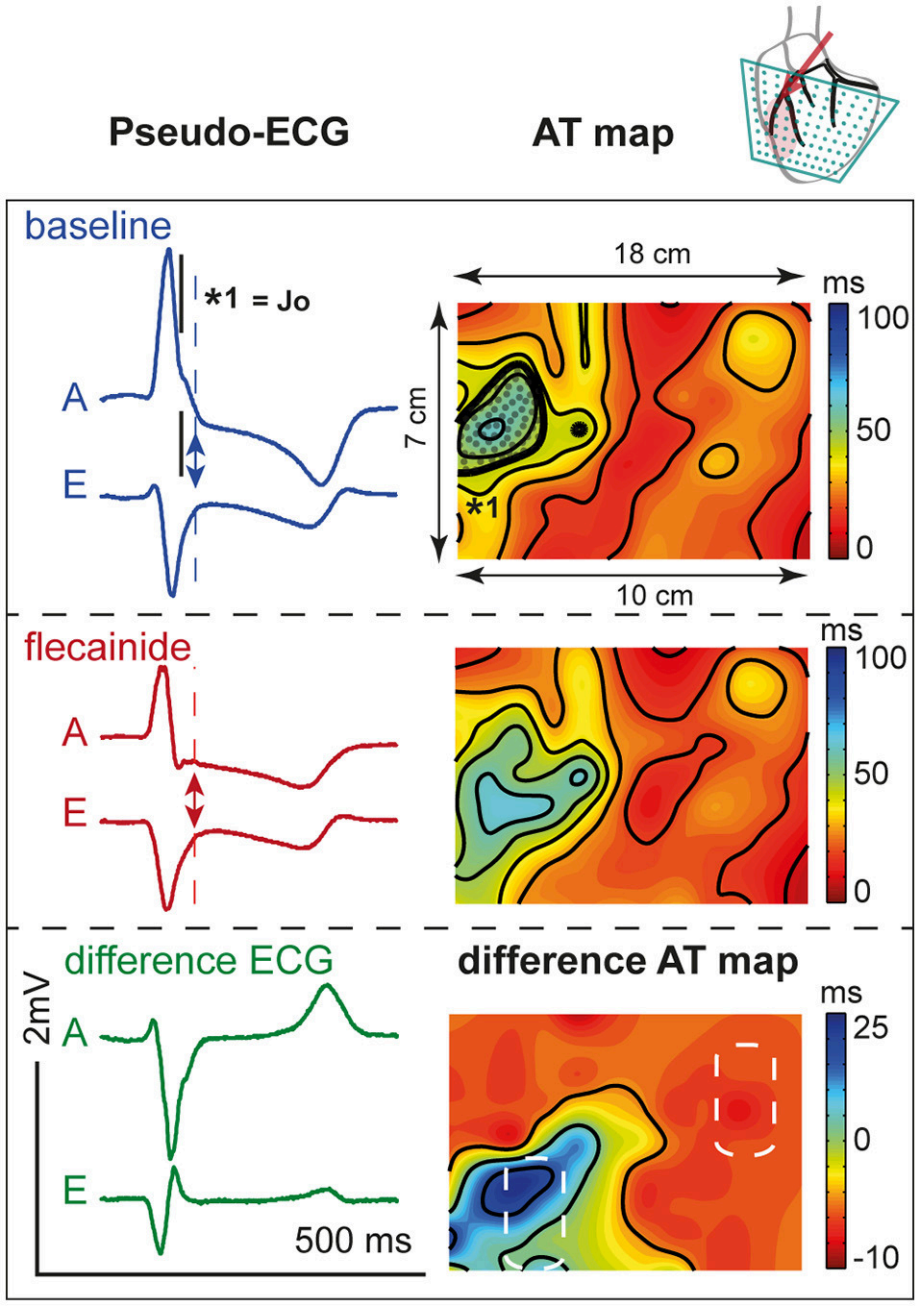
**Activation during**. LAD perfusion. Description is similar to Figure 6. Note that the effect of flecainide (6 μM) was limited to the QRS duration before application and that the region subjected to conduction slowing was relatively early activated before application of flecainide.

Figure 7 shows the activation maps at baseline and during flecainide infusion (6 μM) in the LAD region. This is an extended map with two thirds of the map overlying the LV and one third on the left overlying the RV anterior wall. At baseline, the J-wave interval overlaps that of late activation in the RV (dotted surface) and even outreaches the AT map. After 5 min of flecainide infusion, conduction was delayed in the LAD region and inferolateral J-waves were no longer present. Table 3 demonstrates that within the perfused region (lower left white striped box in Figure 7) activation at baseline was earlier in the activation sequence compared to the region outside the selectively perfused area (right upper box in Figure 7). After flecainide infusion activation was latest in the perfused region.

## Discussion

Our results show that J-waves can be induced as a result of regional conduction slowing due to reduced sodium current only in the lateral region, but not in the anterior or septal region of the heart. Either a regional increase in transient outward potassium current or cellular uncoupling was less effective in inducing J-waves, irrespective of the region. Additionally, a sodium blockade in the rest of the heart attenuated J-waves on the ECG by masking the J-waves in the prolonged QRS.

The experimental data support the simulation data by showing that regional conduction slowing resulting from sodium channel blockade in the lateral but not in the anterior/septal region induces J-waves. The regional conduction slowing in the lateral zone causes local activation to occur beyond the duration of the baseline QRS complex. As a consequence, the J-wave interval coincides largely with the region of conduction delay (Figure 6).

### J-wave mechanism

The mechanism of the inferolateral J-waves is a debated issue (Wellens, 2008; Hoogendijk et al., 2013). The two prevailing hypotheses for inferolateral J-waves are focused either on depolarization (Abe et al., 2010) or repolarization, similar to Brugada Syndrome (Yan and Antzelevitch, 1996). We have shown that the amplitude of the J-point elevation is largest with G_Na_ reduction compared to G_to_ increase and cellular uncoupling. Only G_Na_ reduction in the lateral and anterior region caused J-point elevations that met the criteria of J-waves. This observation supports the depolarization hypothesis. Cellular uncoupling induces smaller J-point elevations (no J-waves). Since activation delays due to G_Na_ reduction and cellular uncoupling were similar (about 15 ms), we argue that their difference in J-point elevations may be explained by the reduced tissue conductivity following cellular uncoupling. In an area of reduced tissue conductivity the current generated by the activation wave front is smaller, resulting in a smaller potential field on the torso. Alternatively, J-waves may be caused by early repolarization of the AP since the increase in G_to_ resulted in minor J-point elevations, which fits with an expected current flow from endocardium toward epicardium during phase one repolarization. However, although the simulated 10-fold increase of G_to_ in this study is very large (see also Supplementary Figure S2) it induces J-point elevations that are only half the size of those induced by G_Na_ reduction, and do not reach the critical J-wave level of 0.1 mV. Also, difference ECGs had smaller amplitudes. The latter hypothesis is therefore the less likely but cannot be excluded based on our data.

Furthermore, it may be argued that I_to_ might play a role in the J-wave induction during a reduction of G_Na_. Indeed, in a model lacking I_to_, the same reduction of G_Na_ led to smaller J-point elevations, although the influence was minor, and might be explained by deeper S-waves already present during baseline (i.e., reference ECG). Moreover, the peak amplitudes of the difference ECGs (G_Na_ minus reference) were 0.06 mV larger in the I_to_ lacking model compared to the model with I_to_ (Supplementary Figure S3B), whereas the timing of the peaks was similar. We therefore conclude that in the simulation of G_Na_ reduction, the role of I_to_ in the J-wave genesis is minor and may even have an opposing effect, i.e., in presence of I_to_ the effects of G_Na_ reduction on the ECG are smaller.

Further amplification of the interventions showed that both G_Na_ reduction to 5% and 20-fold G_to_ increase lead to larger J-wave amplitudes and QRS broadening. However, increasing the G_to_ 20 times does also induce ST-segment elevations in inferolateral leads, which do not resemble the inferolateral J-waves under consideration in this study. Moreover, reduction of G_Na_ to 5% of baseline lead to typical enhancement of the J-wave notching/slurring as seen in patients during the interval preceding an event of ventricular fibrillation (Haïssaguerre et al., 2008).

The amplitude of the difference ECG is largest with G_Na_ reduction followed by cellular uncoupling and then G_to_ increase. Also, the difference ECG was larger with intervention in the anterior region than with intervention in the lateral region, and was smallest with intervention in the septal region. However, the amplitude of the difference ECG is not directly reflected in the amplitude of a J-point elevation. This implies that the timing and spread of the difference ECG contributes importantly to the change in QRS morphology. In the pig experiments, the peak of the difference ECG occurred earlier during LAD perfusion compared to OM perfusion, whereas in the computational results the peak of the difference ECG occurred earlier in the septal compared to the lateral or anterior zone. The separate perfusions in the *ex vivo* hearts, however, did cover slightly different regions within the heart, i.e., with LAD perfusion we influenced the anterior as well as the septal regions. Therefore, combining the computational results from the septal and anterior zones likely represents the *ex vivo* observations more closely. Enhanced G_to_ caused more QRS widening compared to a decreased G_Na_ or cellular uncoupling (Table 1), whereas no activation delay occurred. This indicates that early repolarization does contribute to QRS duration.

Our experimental and simulation data suggest that conduction delay resulting from reduced sodium current in the region that is activated intrinsically late in the activation sequence at baseline, i.e., the lateral region, predominantly contributes to the genesis of inferolateral J-waves, whereas conduction delay in earlier activating regions, i.e., the anterior and/or septal region, shows minor, or no J-point elevations and even J-point depression. We observed that although the AT delay in LAD perfusion was smaller than in OM perfusion it led to J-point depression rather than J-point elevations. We suggest that this difference in J-point deviation is related to the position of the region with delayed activation with respect to the anterolateral recording leads. The septal involvement causes the voltage vector during the J wave to point rightward, away from the anterolateral leads. It is similar to the explanation of how a right bundle branch block results in broad S-wave in V6. The timing of the activation within the QRS complex (normally late activation in the OM region, and normally early activation in the anterior/septal region) causes the LAD region to have a relatively small influence on the J-wave. Recently, it has been shown in a case report that J-waves in the inferior ECG leads can result from delayed activation in the basal inferior LV region, without shortening of the AP duration (Park et al., 2014). This confirms that the region of abnormality is important.

Remarkably, all different interventions—G_Na_ reduction, G_to_ increase, and cellular uncoupling—within the lateral region show similar results in polarity of J-point elevations and difference ECGs, although only G_Na_ reduction led to J-waves. All interventions in the anterior region led to negative difference ECGs in the inferior leads and J-point elevation (no J-waves) were only present in lateral ECG leads. The reason that the effects of interventions are smaller in the anterior and septal zones—despite similar amplitudes of difference ECGs among zones per intervention—is therefore related to the extent of masking by the QRS complex.

### Modulation of J-waves

J-wave amplitude is modulated by heart rate (Aizawa et al., 2012), autonomic tone (Abe et al., 2010), and drugs (Haïssaguerre et al., 2009; Roten et al., 2012). It has been demonstrated in patients that sodium channel blockers like ajmaline, pilsicainide, and flecainide attenuate J-waves in the inferolateral leads (Kawata et al., 2012; Roten et al., 2012; Nakagawa et al., 2014) and broaden the QRS (Roten et al., 2012). Our simulation data explain these findings by demonstrating that G_Na_ reduction in the rest of the heart widens the QRS complex and consequently masks the pre-existing J-waves.

In patients with idiopathic ventricular fibrillation it has been shown that inferolateral J-waves augment after a pause and diminish at higher heart rates (Haïssaguerre et al., 2008; Nakagawa et al., 2014). It has been supposed that the attenuation of J-waves at increasing heart rate results from reduced transient outward potassium current due to the relatively slow recovery from inactivation (Koncz et al., 2014). We did not study the effect of heart rate increase, but we surmise that conduction may also play a role in this phenomenon. Shorter coupling intervals result in lower upstroke velocities of the action potentials, especially in diseased hearts (Kodama et al., 1984). It may therefore have a similar effect as administration of ajmaline and we would expect a concomitant QRS prolongation.

### Study limitations

Nowadays, computational techniques increase in maturity and reliability and are therefore more and more powerful to enlarge our insight in physiology and pathophysiology. As a consequence, it may reduce the number of required animal experiments. However, results from computational models are co-determined by the choice of parameter settings in the model which, due to uncertainty in the experimental data underlying the model, are partly based on assumptions. Due to the assumptions, results from the computational model could not directly be translated to the clinical situation. The results of our additional experiments, however, invigorate the computational findings.

The model did account for interaction between different currents. However, we did not study combinations of factors (G_Na_ reduction, G_to_ increase, and cellular uncoupling), although these together may lead to more exacerbated J-waves, as long as they occur in the same region. Also, it has been shown that different balances of ionic current densities generate viable action potentials (Britton et al., 2013), and therefore it would be of interest to study how the effect of each factor used in this study may change within the setting of different balances of other ion current densities. Yet, our model provides valuable insight into the potential mechanism underlying inferolateral J-waves. Another study limitation is that the pig model lacks I_to_ and therefore it was not suitable to test the role of I_to_. With this model we were, nevertheless, able to induce J-waves, indicating that I_to_ is not required to induce J-waves.

## Conclusion

Conduction slowing caused by reduced sodium current in the lateral region of the heart causes inferolateral J-waves on the ECG. The interval of J-waves coincides with the activation time of the region of delayed activation. The cardiac tissue in which J-waves are induced is characterized by a relatively late activation in the normal heart. Global conduction slowing attenuates J-waves due to masking by the prolonged QRS complex. Enhanced transient outward potassium current and cellular uncoupling have minor potency to elicit inferolateral J-waves. Although our study cannot exclude the role of repolarization abnormality, it predominantly affirms the depolarization hypothesis especially when tissue conductivity is preserved. Our study also provides an explanation for J-wave attenuation by sodium channel inhibition.

## Author contributions

VM: design, data acquisition, data analyses, data interpretation, writing MP: design, data acquisition, data analyses, data interpretation, writing CC: data interpretation, revising CB: data acquisition, revising JDB: data interpretation, revising RC: design, data acquisition, data interpretation, revising. All authors meet the following criteria: (i) Substantial contributions to the conception or design of the work; or the acquisition, analysis, or interpretation of data for the work, (2) Drafting the work or revising it critically for important intellectual content, (3) Final approval of the version to be published, (4) Agreement to be accountable for all aspects of the work in ensuring that questions related to the accuracy or integrity of any part of the work are appropriately investigated and resolved.

## Funding

Support from Fundació Marató de TV3 grant (project 080632), Barcelona, Spain and from Fondation LeDucq grant (ShapeHeart, project 10801). This work was granted access to the HPC resources of IDRIS under the allocation x2015037379 made by GENCI.

### Conflict of interest statement

The authors declare that the research was conducted in the absence of any commercial or financial relationships that could be construed as a potential conflict of interest.

## Abbreviations

AT: activation time
ER: Early Repolarization
LAD: left anterior descending coronary artery
OM: left obtuse marginal coronary artery
pseudo-ECG: pseudo-electrocardiogram
