# Supplementary material for: Reduced Sodium Current in the Lateral Ventricular Wall Induces Inferolateral J-Waves
Source: Front Physiol. 2016 Aug 26;7:365. doi: 10.3389/fphys.2016.00365 (PMC5000556; doi:10.3389/fphys.2016.00365)
Supplement: Supplementary file 1 [file DataSheet1.PDF]

Supplementary Material

Regional conduction slowing induces inferolateral J-waves

Veronique M. F. Meijborg PhD[1,2,#], Mark Potse PhD[3,4,5,#], Chantal E. Conrath MD PhD[1], Charly N. W. Belterman VA[1,3], Jacques M. T. de Bakker PhD[1,2,6], Ruben Coronel MD PhD[1,3]

*** Correspondence: Corresponding Author: veromeijborg@gmail.com**

Supplementary Methods

Computational model

A whole-heart monodomain reaction-diffusion model at 0.2 mm spatial resolution (*dx*) and 10 to 50 µs temporal resolution (*dt*) was used to simulate propagating action potentials throughout the cardiac tissue.(Potse et al., 2006) This model relates the transmembrane ionic current to the membrane potential on a macroscopic scale. Ionic currents within the heart were computed using a membrane model of the human ventricular myocyte including the differential characteristics of subendocardial (50%), midmyocardial (30%), and subepicardial myocytes (20%).(Ten Tusscher et al., 2004)

The model anatomy (heart and torso) was based on MRI data of a subject with normal cardiac anatomy. From these data a three-dimensional finite-difference mesh was created. The transmural fiber rotation was incorporated using a rule-based method.(Krause et al., 2012; Potse et al., 2006) The heart model was embedded in an inhomogeneous human torso model (*dx*= 1 mm), including lungs and intracavitary blood volumes. The surface ECG was computed (*dt*= 1 ms) by solving the potential field in the heart and torso (including comprising lungs, intracavitary blood masses, and an anisotropic skeletal muscle layer) resulting from the computed transmembrane current densities.(Potse et al., 2009)

Experimental setup

Pigs (n=2, male, weight 50-60 kg) were premedicated with ketamine (10-15 mg/kg, Nimatek, Eurovet Animal Health BV) and midazolam (1-2 mg/kg, Actavis, Iceland) intramuscularly, and anesthetized with 15 mg/kg pentobarbital (Nembutal, Ceva Santé Animale) intravenously. The animals were intubated and ventilated with room air and oxygen plus isoflurane (1:1 + 1.5%). After a midsternal thoracotomy, 5000 international units of heparin (Leo Pharma) were injected intravenously. We infused Tyrode’s solution intravenously and simultaneously collected 4 to 5 L diluted blood. The heart was excised and perfused according to Langendorff. The left obtuse marginal coronary artery (OM) or left anterior descending coronary artery (LAD) was dissected over a distance of 5 mm and a ligature was passed underneath it. A cannula was introduced via a small incision into the OM or LAD and fixed by tying the ligature. The cannula was connected to a separate perfusion system with the same blood-Tyrode’s mixture.

Electrophysiologic recordings

We used 2 different electrode grids for recordings of the local unipolar epicardial electrograms recorded against a reference electrode at the aortic root. In the heart with the OM perfusion we used an 11x11 electrode grid with an interelectrode distance of 5 mm. In the heart with the LAD perfusion we used a 9x12 electrode grid with an interelectrode distance of 9 mm vertically, and of 9 – 17 mm horizontally. Supplementary Figure S1 shows a schematic view of the two electrode grid configurations.

Supplementary Results

Baseline simulations

At baseline, earliest activation was in the septal zone (29±8 ms), followed by the lateral zone (40±8 ms) and 3 ms later the anterior zone (43±7 ms). Total activation time was 77 ms and latest activation occurred outside the 3 predefined zones.

Action potential morphologies after interventions.

Supplementary Figure S2 shows the resulting action potential (AP) morphologies transmurally in the affected zone and the epicardial AP morphology before and after intervention. G_{Na} reduction to 12.5% in the affected zone led to a delay in activation without affecting the AP morphology (Supplementary Figure S2A). A 10-fold G_{to} increase caused in the affected region a deeper AP notch at the epicardium, without influencing the endocardial AP notch. The endocardial layer did have minor I_{to} and therefore lacks a clear notch preceding the plateau phase (Supplementary Figure S2B). Cellular uncoupling in the affected region caused a delay of the AP without affecting the AP morphology (Supplementary Figure S2C).

Simulation of regional G_{Na} reduction

After G_{Na} reduction in the lateral zone the difference ECG (lateral G_{Na} reduction – baseline) had mainly positive amplitudes occurring during the late phase of the QRS complex (Figure 2A, Table 1). After G_{Na} reduction in the anterior zone the difference ECG had positive amplitudes in the lateral leads (I, aVL, and V4-V6) and negative amplitudes in the inferior leads (II,III,aVF), also occurring in the late phase of the QRS (Figure 2B, Table 1). After G_{Na} reduction in the septal zone the difference ECG (septal G_{Na} reduction – baseline) showed minor fractionated differences mainly of negative amplitude and halfway the QRS complex (Figure 2C, Table 1).

Simulation of regional G_{to} increase

G_{to} increase in the lateral region induced J-point elevations in 4 of the 12 ECG leads (I,II,III and V6) with a maximum amplitude of 0.07 mV in lead V6. The difference ECG showed low-amplitude and generally positive differences during the late phase of the QRS. In the anterior region, G_{to} increase induced J-point elevations in 3 lateral leads (notch in I and V5 and slur in aVL) but led to J-point depression in the inferior leads. Difference ECGs had somewhat larger amplitudes (positive in lateral leads, negative in inferior leads) and larger QRS prolongation compared to the lateral G_{to} increase. G_{to} increase in the septal region did not induce J-point elevations. Also, difference ECGs were small, fractionated, and located halfway the QRS. Overall, G_{to} increase did prolong the QRS duration, but did not induce an activation delay in the affected zone or in the unaffected zones.

Simulation of cellular uncoupling

Reduction of the intercellular coupling in the lateral zone cause small J-point notching in lead II and lead V6. Differences in ECGs occurred generally during the late phase of the QRS and the amplitudes were positive in lateral leads and negative in inferior leads. The amplitude of the difference ECGs were in between the amplitudes observed in G_{Na} reduction and G_{to} increase. In the anterior zone uncoupling caused J-point notching in lead I and aVL with higher amplitudes than uncoupling in the lateral zone. The difference ECGs show smaller, but wider, differences. Again, uncoupling in the septal zone did not induce J-point elevations and the difference ECGs were small and fractionated during the total QRS duration. Although – similar to G_{Na} reduction – cellular uncoupling induced activation delays of about 15 ms and caused maximum activation in the affected zone that correspond with end of QRS, J-point elevations were smaller compared to G_{Na} reduction.

Baseline simulation without I_{to}

To evaluate the role of I_{to} in the model we also created a similar model lacking I_{to} ($G_{to} = 0$). In the reference ECG of the model without I_{to}, the QRS morphology differs slightly from that in the baseline ECG of the ‘normal model’ with I_{to} (Supplementary Figure S3A). The inferior leads showed a smaller R-wave amplitude and the inferior and precordial leads showed a larger S-wave amplitude, without changing the QRS duration. After G_{Na} reduction (to 12.5% of baseline) in this ‘no I_{to} model’, QRS duration was equally broadened and the difference ECGs showed a 0.06 mV larger peak amplitude with the same timing compared to the G_{Na} reduction in the ‘normal model’ with I_{to} (Supplementary Figure S3B).

Simulation of amplified interventions

Supplementary Figure S4 shows the results of G_{Na} reduction to 5% of baseline and G_{to} increase of 20-fold of the baseline of the ‘normal model’. These simulations were compared to the reference ECG of the model lacking I_{to}. After reduction of G_{Na} to 5%, J-wave amplitudes and QRS duration were further increased (Supplementary Figure S4B) when compared to J-waves after a reduction to 12.5% G_{Na}. The peak amplitude of the difference ECG did increase, although this was only 0.05 mV. The timing of the peak of the difference ECG did not change.

After a 20-fold increase of G_{to}, J-wave amplitudes increased and the QRS duration doubled but the latter may also be skewed by the difficulty to measure it because of the ST-elevation (Supplementary Figure S4B) when compared to a 10-fold G_{to} increase in a ‘normal model’. The peak amplitude of the difference ECG did increase 3 to 4-fold and the timing of the peak was 7 ms later.

Experiments: pseudo-ECGs

The timing of Jo after flecainide infusion in the OM differed among pseudo-ECG leads and was earliest in inferior leads and later in superior leads (first moment of Jo given in Table 2). The first moment of Jo in the inferolateral leads was earlier after flecainide infusion than at baseline.

Supplementary References

Krause, D., Potse, M., Dickopf, T., Krause, R., Auricchio, A., and Prinzen, F. W. (2012).

"Hybrid Parallelization of a Large-Scale Heart Model," in *Facing the Multicore - Challenge II; Aspects of New Paradigms and Technologies in Parallel Computing.*, eds. R. Keller, D. Kramer, and J. P. Weiss (Springer Heidelberg), 120–132. doi:10.1007/978-3-642-30397-5_11.

Potse, M., Dubé, B., Richer, J., Vinet, A., and Gulrajani, R. M. (2006). A comparison of monodomain and bidomain reaction-diffusion models for action potential propagation in the human heart. *IEEE Trans. Biomed. Eng.* 53, 2425–35. doi:10.1109/TBME.2006.880875.

Potse, M., Dubé, B., and Vinet, A. (2009). Cardiac anisotropy in boundary-element models for the electrocardiogram. *Med. Biol. Eng. Comput.* 47, 719–29. doi:10.1007/s11517-009-0472-x.

Ten Tusscher, K. H. W. J., Noble, D., Noble, P. J., and Panfilov, A. V (2004). A model for human ventricular tissue. *Am. J. Physiol. Heart Circ. Physiol.* 286, H1573–89. doi:10.1152/ajpheart.00794.2003.

Supplementary Figures

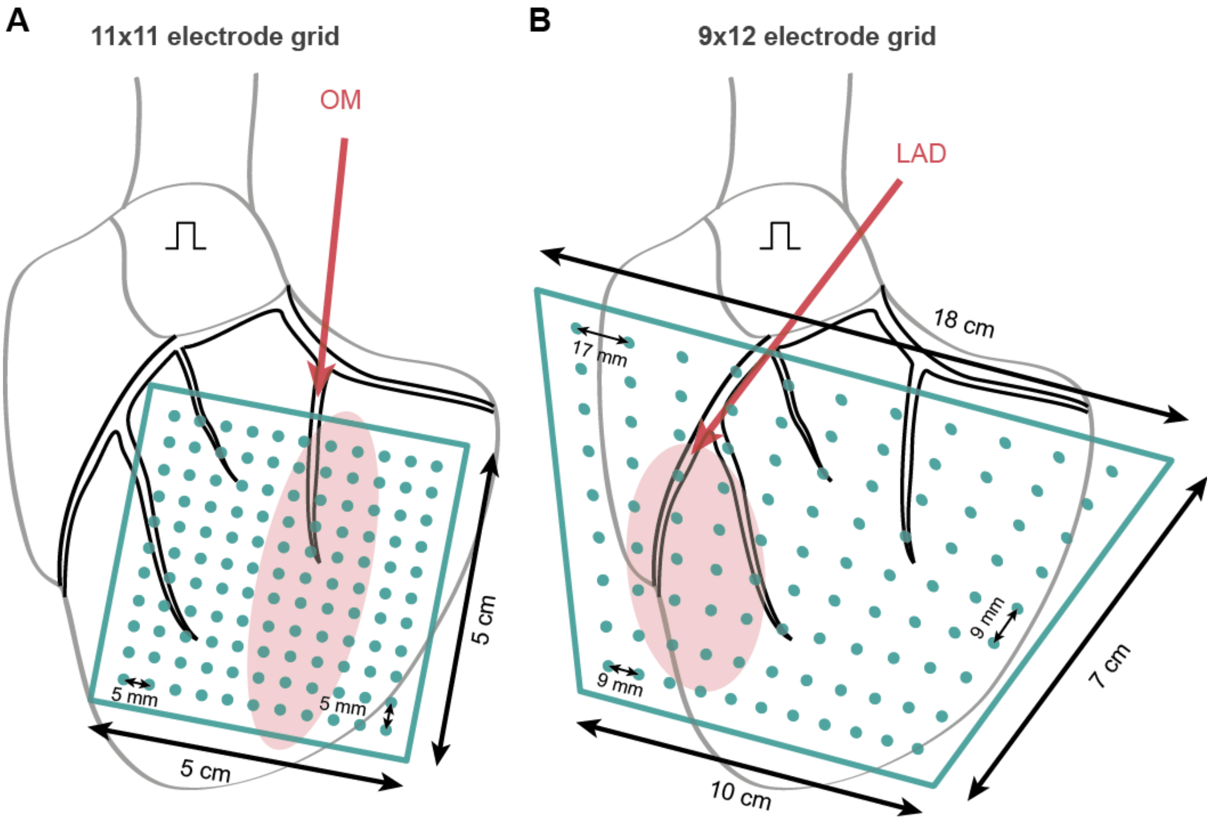

Supplementary Figure S1: Schematic view of the electrode grid configuration in both Langendorff-perfused hearts. **A:** 11x11 electrode grid in heart with OM perfusion, and **B:** 9x12 electrode grid in heart with LAD perfusion. Red shaded area indicates the selectively perfused tissue and the red arrows indicate the position of the cannula.

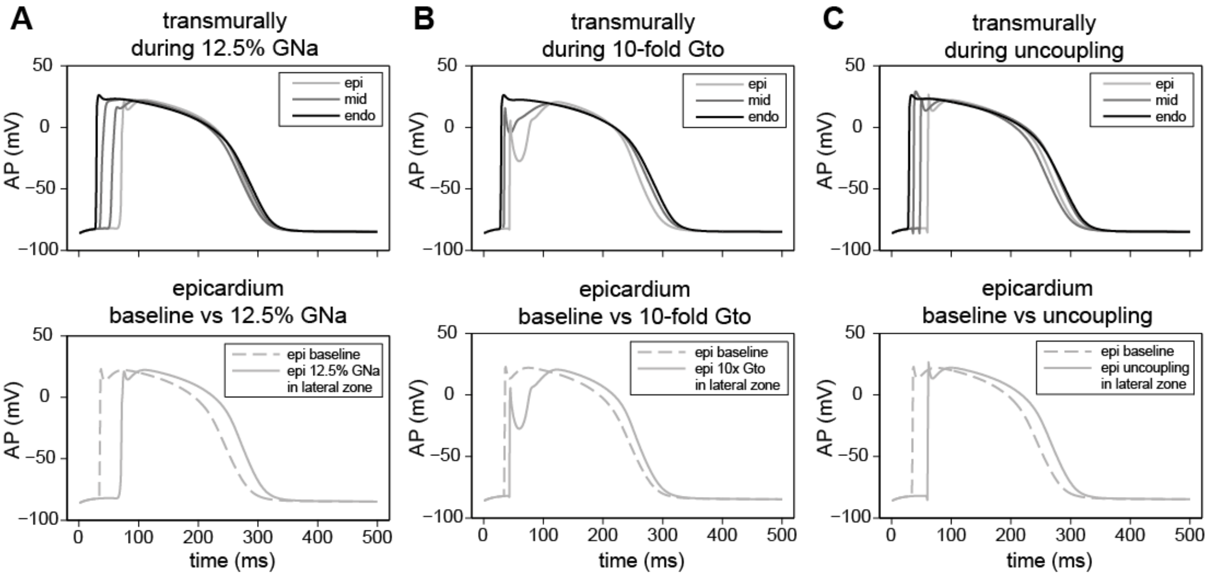

Supplementary Figure S2: Simulated action potential (AP) morphologies in the affected zone. **Top panels:** APs transmurally during intervention. **Bottom panels:** epicardial AP during intervention compared to baseline. **A:** after G_{Na} reduction in the lateral zone (note the activation delay without affecting the AP morphologies) **B:** after 10-fold G_{to} increase in the lateral zone (note the deep notch of the epicardial action potential compared to the endocardial action potential) **C:** after cellular uncoupling in the lateral zone (note the activation delay without affecting the AP morphologies).

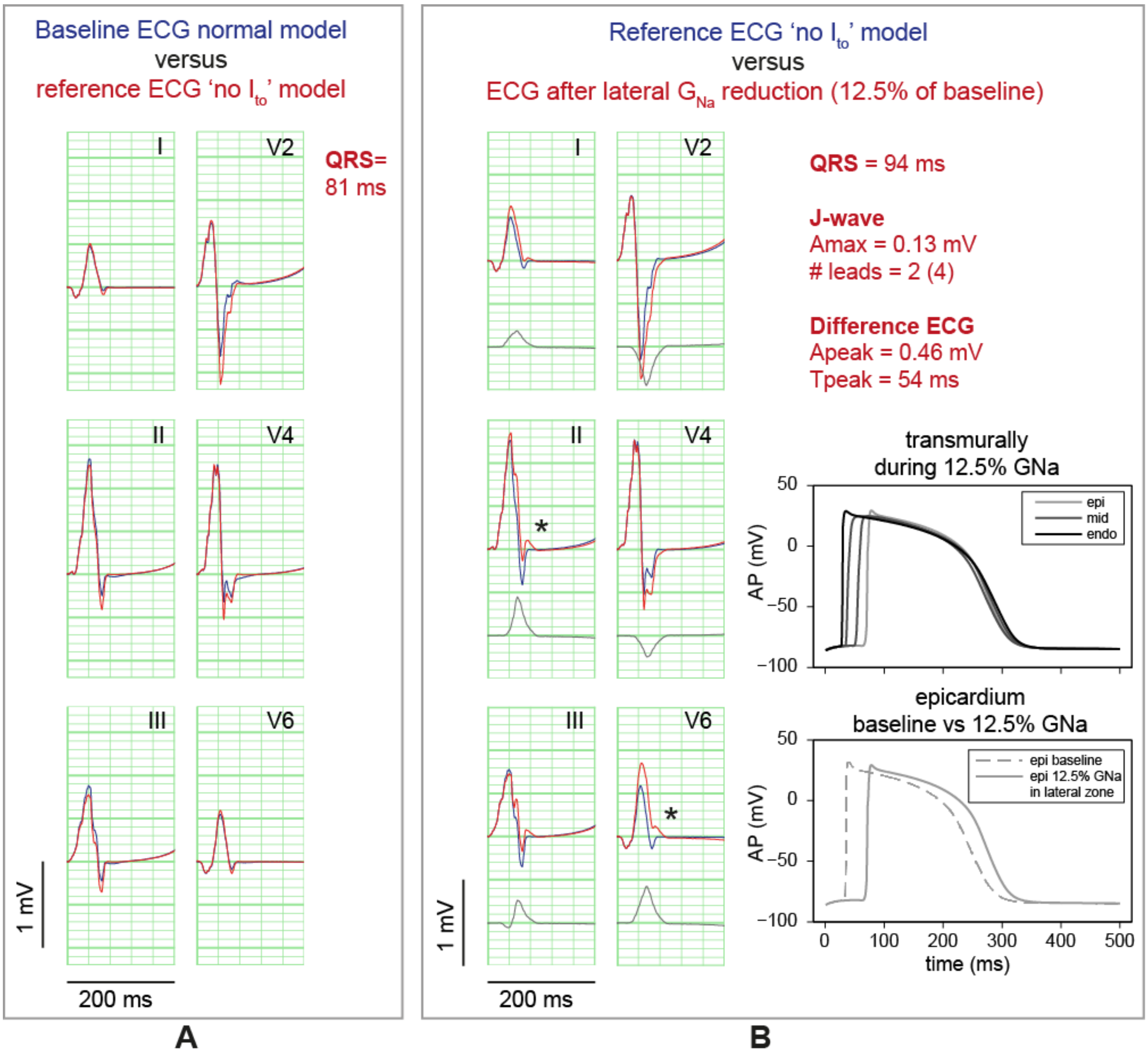

Supplementary Figure S3: A: Comparison of the simulated ECGs from a normal model (*blue*) versus a model lacking I_{to} (*red*). Note that the loss of I_{to} leads to somewhat smaller R-wave amplitudes and somewhat larger S-wave amplitudes. **B:** Reference ECG of 'no I_{to}' model (*blue*) and ECG resulting from G_{Na} reduction to 12.5% of baseline in the lateral zone (*red*). *Gray*: Difference ECG (G_{Na} reduction minus baseline). Asterisks= J-waves. Also the simulated action potential (AP) morphologies in the affected zone are shown. **Top panel:** transmural APs during intervention. **Bottom panel:** epicardial AP during intervention compared to baseline.

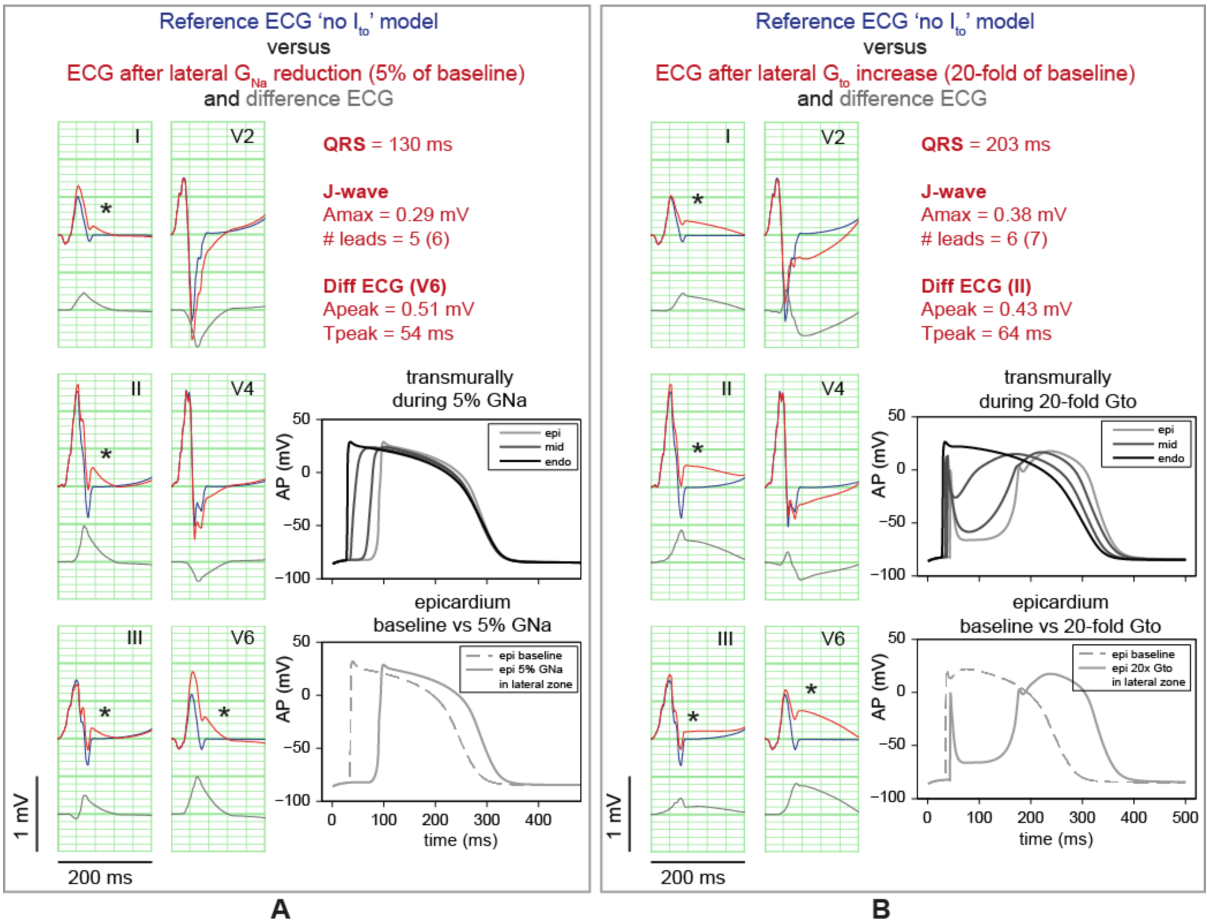

Supplementary Figure S4: Reference ECGs of 'no I_{to}' model (*blue* in **A** and **B**) and ECG resulting from G_{Na} reduction to 5% of baseline (*red* in **A**) or from a 20-fold increase in G_{to} (*red* in **B**) in the lateral zone. Note that J-waves become larger in both interventions, although in the G_{to} increase also ST-segment elevations and T-wave inversion occur (only QRS is shown). *Gray*: Difference ECG (intervention minus baseline). Asterisks= J-waves. Also the simulated action potential (AP) morphologies in the affected zone are shown. **Top panel:** transmural APs during intervention. **Bottom panel:** epicardial AP during intervention compared to baseline. Note that during a 20-fold G_{to} increase there is a loss of AP dome at the epicardium followed by an AP dome resembling an after depolarization.
